# Impact of an antimicrobial stewardship in a 126-bed community hospital with close communication between pharmacists working on post-prescription audit, ward pharmacists, and the antimicrobial stewardship team

**DOI:** 10.1186/s40780-021-00206-x

**Published:** 2021-08-01

**Authors:** Satoshi Nakamura, Takashi Arima, Ryoichi Tashiro, Satomi Yasumizu, Hayato Aikou, Emi Watanabe, Takashi Nakashima, Yuho Nagatomo, Ikuyo Kakimoto, Toshiro Motoya

**Affiliations:** 1 Infection Control Team and Antimicrobial Stewardship Team, Tarumizu Chuo Hospital, Tarumizu Municipal Medical Center, Tarumizu, Japan; 2 Division of Pharmacy, Tarumizu Chuo Hospital, Tarumizu Municipal Medical Center, Tarumizu, Japan; 3 Division of Urology, Tarumizu Chuo Hospital, Tarumizu Municipal Medical Center, Tarumizu, Japan; 4 Department of Nursing, Tarumizu Chuo Hospital, Tarumizu Municipal Medical Center, Tarumizu, Japan; 5 Division of Clinical Laboratory, Tarumizu Chuo Hospital, Tarumizu Municipal Medical Center, Tarumizu, Japan

**Keywords:** Antimicrobial stewardship, Antimicrobial stewardship team, Appropriate use of antimicrobials, Culture examination, De-escalation, Antimicrobial use density, Days of therapy, 30-day mortality, 30-day recurrence rate, Pharmacist

## Abstract

**Background:**

Antimicrobial stewardship (AS) is defined as coordinated interventions to improve and measure the appropriate use of antimicrobial agents. However, available resources for AS differ depending on the size of the clinical setting. Therefore, AS programs based on guidelines need to be selected in order to implement AS in small- to medium-sized hospitals. The present study compared the impact of AS in a 126-bed community hospital between pre- and post-AS periods.

**Methods:**

The present study was retrospectively performed by selecting data on eligible patients from electronic medical records stored in the central database of the hospital. The roles of the AS team included weekly rounds and recommendations on the appropriate use of antimicrobials, and pharmacists working on post-prescription audits and pharmaceutical care at the bedside closely communicated with the AS team to assist with its implementation. As process measurements, the order rate of culture examinations, the conducting rate of de-escalation, antimicrobial use density (AUD), days of therapy (DOT), and the AUD/DOT ratio of carbapenems and tazobactam-piperacillin (TAZ/PIPC) were measured. Thirty-day mortality and recurrence rates were examined as clinical outcomes.

**Results:**

A total of 535 patients (288 in the pre-AS period and 247 in the post-AS period) were enrolled in the present study. The recommendation rate to prescribers significantly increased (*p* < 0.01) from 10.4% in the pre-AS period to 21.1% in the post-AS period. The order rate of culture examinations increased from 56.3 to 73.3% (*p* < 0.01). The conducting rate of de-escalation increased from 10.2 to 30.8% (*p* < 0.05). The AUD of carbapenems and TAZ/PIPC significantly decreased (*p* < 0.05). The DOT of carbapenems (*p* < 0.01) and TAZ/PIPC (*p* < 0.05) also significantly decreased. The AUD/DOT ratio of carbapenem significantly increased from 0.37 to 0.60 (*p* < 0.01). Thirty-day mortality rates were 11.2 and 14.2%, respectively, and were not significantly different. The 30-day recurrence rate significantly decreased (*p* < 0.05) from 14.7 to 7.5%.

**Conclusions:**

The implementation of AS in this hospital improved the appropriate use of antimicrobials without negatively affecting clinical outcomes. These results may be attributed to close communication between pharmacists working on post-prescription audits and pharmaceutical care at the bedside and the AS team.

## Background

Antimicrobial resistance (AMR) increases the difficulties associated with treating an infectious disease and is a serious threat to human health. It has resulted in longer hospital stays, higher hospital costs, and greater mortality [1, 2]. The inappropriate use of antimicrobial agents is considered to be a major factor increasing AMR, and antimicrobial stewardship (AS) is defined as coordinated interventions to improve and measure the appropriate use of antimicrobial agents. AS has been shown to decrease antimicrobial use density (AUD), days of therapy (DOT), and in-hospital mortality and also contributes to cost savings [3–5].

AS is a medical stuff function that optimizes clinical outcomes while minimizing the unintended consequences of antimicrobial use, and includes the appropriate selection, dosing, route, and duration of antimicrobial therapy [6]. Recommended core members of the AS team (AST) include an infectious diseases physician, a clinical pharmacist with infectious disease training, a clinical microbiologist, an information system specialist, and an infection control professional. Comprehensive AS programs (ASPs) that include a number of elements, such as interventions, optimization, microbiology and laboratory diagnostics, measurements, special populations, and education, have been proposed as therapeutic guidelines to combat AMR [6, 7]. On the other hand, the conditions under which AS is implemented differ based on local antimicrobial use and resistance. Furthermore, available resources including manpower, on-site microbiology laboratories, and AST organizations differ depending on the size of the institution or clinical setting. According to a nationwide survey conducted by the Japanese Society of Chemotherapy, small- to medium-sized hospitals had fewer on-site microbiology laboratories, AST organizations, pharmacists and collaborations with ward pharmacists than large-sized hospitals [8, 9]. Therefore, ASPs according to the guidelines need to be selected to implement AS in small- to medium-sized hospitals [6, 7]. To date, ASPs have been widely implemented in large-sized hospitals. Few studies have examined the implementation of AS in the small- to medium-sized hospitals [10–12], and the impact of AS on the use of antimicrobials and clinical outcomes in hospitals with finite resources is limited. Accordingly, further studies are needed on the strategies to improve the efficacy of AS in small- to medium-sized hospitals.

Tarumizu Chuo hospital is a 126-bed community hospital. The Infection Control Team (ICT) of the hospital include a physician (Infection Control Doctor: ICD), a clinical pharmacist without authorized infectious disease training, a nurse, and a clinical laboratory technologist, who concurrently have been members of AST since June 2019. Prior to the organization of AST, antimicrobial agents were administered at the discretion of the respective attending physicians. At that time, carbapenem or Tazobactam-Piperacillin (TAZ/PIPC) was administered to some cases as an empiric treatment for severe infectious diseases. Furthermore, antimicrobial agents were prescribed over long periods to some cases in which causative microorganisms were not identified. To improve these conditions, AST was organized in June 2019, and started weekly rounds and prospective audits of antimicrobial use with direct interactions and feedback. Regarding the prospective audit of antimicrobial use, pharmacists including an AST pharmacist working on post-prescription audits also started to check medical records in detail every day and also collected information on the conditions of patients as ward pharmacists. Pharmacists other than the AST pharmacist closely communicated with the AST pharmacist and supported the implementation of AS.

Therefore, the present study was performed to assess the impact of AS on the use of antimicrobials and clinical outcomes at this 126-bed community hospital with close communication between pharmacists working on post-prescription audit, ward pharmacists, and AST.

## Methods

### Study design

The present study was conducted at Tarumizu Chuo Hospital, Tarumizu Municipal Medical Center. Study periods were the pre-AS period (between July 1 and December 31, 2018) and post-AS period (between July 1 and December 31, 2019). Eligible patients were inpatients treated with specific antimicrobials, administered parenteral antimicrobials for more than 14 days, with antimicrobial-resistant pathogens, and with a microbial-positive blood culture. Specific antimicrobials were meropenem (MEPM), imipenem/cilastatin (IPM/CS), TAZ/PIPC, levofloxacin, cefepime, cefozopran, vancomycin, and linezolid. Antimicrobial-resistant pathogens included methicillin-resistant *Staphylococcus aureus* (MRSA), extended-spectrum β-lactamase-producing enterobacteria (ESBL-producing enterobacteria), carbapenem-resistant *Enterobacteriaceae* (CRE), vancomycin-resistant enterococci (VRE), multidrug-resistant *Acinetobacter* spp. (MDRA), multidrug-resistant *Pseudomonas aeruginosa* (MDRP). MDRA and MDRP were defined as strains that were resistant to carbapenems (minimum inhibitory concentration: MIC ≥16 mg/L), fluoroquinolones (MIC ≥4 mg/L), and amikacin (MIC ≥32 mg/L) based on the criteria for MDR strains specified by the Law Concerning the Prevention of Infections and Medical Care for Patients with Infections by the Japanese Ministry of Health and Welfare [13]. Patients treated with specific antimicrobials and parenteral antibiotics for more than 14 days were extracted by the AST pharmacist, while those with antimicrobial-resistant pathogens and a microbial-positive blood culture were extracted by the AST clinical laboratory technologist before AST rounds.

AS implementation in the pre-AS period included the registration system of specific antimicrobials when used, the monthly monitoring of antimicrobial consumption, the monthly monitoring of antimicrobial resistance, and annual lectures for hospital staff and occasional discussions with an attending physician. These activities were continued in the post-AS period. Regarding the post-prescription audit of antimicrobial use, dose adjustments based on the package inserts of medicines were implemented in the pre-AS period. Furthermore, careful checks of medical records, including prescriptions, laboratory test values, vital signs, and other conditions for eligible patients, were performed daily by pharmacists in the post-AS period. Pharmacists other than the AS pharmacist then closely communicated with the AST pharmacist and recommendations to prescribers from AST were implemented. Details on recommendations by AST to prescribers are shown in Table 1 and include the following: 1) Order of culture examinations, 2) Selection of antimicrobials, 3) Dose of antimicrobials, 4) Duration of antimicrobial therapy, 5) De-escalation, 6) Therapeutic drug monitoring, and 7) Others. Rounds by AST had not been implemented in the pre-AS period; however, weekly rounds were performed in the post-AS period. Ward pharmacists also collected information on the conditions of patients. They closely communicated with the AST pharmacist and supported the implementation of AS. Regarding the ward activity of pharmacists, the number of cases claiming the drug management guidance fee, one of the medical fees for pharmaceutical care by ward pharmacists, increased from 321 in the pre-AS period to 683 in the post-AS period. This increase reflected rounds being more frequently performed by ward pharmacists. The AST pharmacist typically integrated patient information on pharmacotherapy, corresponded for inquiries including the selection of antibiotics from prescribers, and made recommendations for the appropriate use of antibiotics where necessary. The frequency of recommendations was higher in the post-AS period than in the pre-AS period. Furthermore, the AST pharmacist typically made hospital antibiograms and provided data to prescribers and other medical staff in order to ensure the appropriate use of antimicrobials in the post-AS period, in contrast to the pre-AS period.

**Table 1:** Recommendations to prescribers from the antimicrobial stewardship team

1)	Order of culture examinations	・Identification of microbial pathogens and implementation of susceptibility tests before administration of parenteral antimicrobials.
2)	Selection of antimicrobials	・Selection of antimicrobials for empirical initial antimicrobial therapy. ・Changes in antimicrobials based on the results of susceptibility tests (not including de-escalation).
3)	Dose of antimicrobials	・Dose optimization based on the severity of disease and the renal function of patients.
4)	Duration of antimicrobial therapy	・Recommendation to discontinue antimicrobial therapy.
5)	De-escalation	・Change in excessively broad therapy to more targeted antimicrobial therapy.
6)	Therapeutic drug monitoring	・Dose and/or dosing interval recommendation based on the results of a pharmacokinetic analysis.
7)	Others	・Other recommendations concerning infectious disease therapy.

The present study was retrospectively performed by selecting data on eligible patients from electronic medical records stored in the central database of the hospital.

### Monitoring of process measurements

Regarding process measurements of the appropriate use of antimicrobials, the order rate of culture examinations to identify pathogens and the conducting rate of de-escalation were examined and expressed as follows:

$$ \mathrm{Order}\ \mathrm{rate}\ \mathrm{of}\ \mathrm{culture}\ \mathrm{examinations}\ \left(\%\right)=\left(\mathrm{The}\ \mathrm{number}\ \mathrm{of}\ \mathrm{patients}\ \mathrm{for}\ \mathrm{whom}\ \mathrm{culture}\mathrm{s}\ \mathrm{were}\ \mathrm{ordered}/\mathrm{All}\ \mathrm{participating}\ \mathrm{patients}\right)\times 100 $$

$$ \mathrm{Conducting}\ \mathrm{rate}\ \mathrm{of}\ \mathrm{de}-\mathrm{escalation}\ \left(\%\right)=\left(\mathrm{The}\ \mathrm{number}\ \mathrm{of}\ \mathrm{patients}\ \mathrm{for}\ \mathrm{whom}\ \mathrm{de}-\mathrm{escalation}\ \mathrm{was}\ \mathrm{conducted}/\mathrm{All}\ \mathrm{patients}\ \mathrm{using}\ \mathrm{specific}\ \mathrm{antibiotics}\right)\times 100 $$

We also examined AUD, DOT, and the AUD/DOT ratio of carbapenems (MEPM and IPM/CS) and TAZ/PIPC, which were more frequently used among the specific antimicrobials at this hospital. AUD and DOT were expressed as follows:

$$ \mathrm{AUD}\ \left(\mathrm{DDDs}/\mathrm{1,000}\ \mathrm{patient}-\mathrm{days}\right)=\left[\mathrm{Total}\ \mathrm{dose}\ \left(\mathrm{g}\right)\ \mathrm{of}\ \mathrm{antimicrobial}\ \mathrm{used}/\mathrm{DDD}\ \mathrm{x}\kern0.5em \mathrm{Total}\ \mathrm{days}\ \mathrm{of}\ \mathrm{hospital}\ \mathrm{stay}\right]\times 1000 $$

$$ \mathrm{DOT}\ \left(\mathrm{DOTs}/1,000\ \mathrm{patient}-\mathrm{days}\right)=\left[\mathrm{Total}\ \mathrm{days}\ \left(\mathrm{day}\right)\ \mathrm{antimicrobials}\ \mathrm{were}\ \mathrm{used}/\mathrm{Total}\ \mathrm{days}\ \mathrm{of}\ \mathrm{hospital}\ \mathrm{stay}\right]\times 1000 $$

The defined daily dose (DDD) was the value defined by the World Health Organization.

### Clinical outcome

We examined 30-day mortality and recurrence rates as the clinical outcomes of AS. The 30-day mortality rate was calculated as the number of patients who died within 30 days of the initiation of antimicrobial treatment divided by all participating patients. The 30-day recurrence rate was calculated as the number of patients with recurrence of the same disease within 30 days of discontinuing antimicrobial therapy divided by all participating patients.

### Statistical analysis

Statistical analyses were performed using ystat2018.xls for Windows (Igakutosyosyuppan co., Ltd., Tokyo, Japan). Nominal variables of the two groups were compared using the Chi-squared test and continuous variables using the Mann-Whitney U-test. A *p*-values of < 0.05 was considered to be significant.

### Ethics approval and consent to participate

The present study was approved by the Ethics Committee of Tarumizu Chuo Hospital (No. 20–1). The Ethics Committee waived the need for informed consent since this study used anonymous, aggregate, retrospective data.

## Results

### Patient characteristics

A total of 535 patients were enrolled in the present study. The pre-AS and post-AS groups included 288 and 247 patients, respectively. The characteristics of patients enrolled in the present study are shown in Table 2. No significant differences were observed in age, sex, body weight, renal function, or infected organs between the groups. Among infected organs, the ratio of respiratory organs was the highest in both groups, but was not significantly different.

**Table 2:** Patient characteristics

	pre-AS period	post-AS period	*p*-value
	(*n* = 288)	(*n* = 247)	
Age (years)	81.9 ± 13.8	82.9 ± 12.2	0.281
Sex (F/M)	129/159	111/136	0.973
Body weight (Kg)	47.0 ± 13.8	46.8 ± 14.3	0.435
CCr (mL/min)	46.2 ± 30.7	45.4 ± 30.0	0.363
Focus of infection			
Respiratory	148 (51)	152 (62)	0.212
Urinary tract	75 (26)	44 (18)	0.068
Gastrointestinal and Intraabdominal	38 (13)	27 (11)	0.479
Bone and soft tissue	17 (6)	13 (5)	0.762
Others	10 (4)	12 (5)	0.440

Values are presented as means±SD or numbers (%), *CCr* Creatinine clearance

Data were analyzed using the Mann-Whitney U-test or chi-squared test

### AS activity

Details on recommendations by the AST for prescribers are shown in Table 3. The total number of patients recommended was 30 (10.4%) in the pre-AS period and 52 (21.1%) in the post-AS period. The post-AS period showed a significantly higher rate (*p* < 0.01). The number of cases recommended were 30 and 68, respectively. In the post-AS period, some patients received two or more recommendations. The acceptance rate of recommendations by prescribers was 93.3% (28/30) in the pre-AS period and 94.1% (64/68) in the post-AS period, which was not significantly different. The number of recommendations on the selection of antimicrobials increased the most in the post-AS period. The numbers of recommendations for de-escalation (7) and the duration of antimicrobial therapy (4) in the post-AS period increased from 0 cases in the pre-AS period.

**Table 3:** Recommendations to subscribers from the antimicrobial stewardship team

	pre-AS period	post-AS period	*p*-value
	(*n* = 288)	(*n* = 247)	
Order of culture examinations	1	4	
Selection of antimicrobials	4	26	
Dose of antimicrobials	18	20	
Duration of antimicrobial therapy	0	4	
De-escalation	0	7	
Therapeutic drug monitoring	3	5	
Others	4	2	
Total recommendations (cases)	30	68	
Applicable patients (persons)	30	52	
Recommendation rate	10.4 (30/288)	21.1 (52/247)	< 0.01
Acceptance rate of recommendation	93.3 (28/30)	94.1 (64/68)	1.00

Values are presented as numbers or % (Number of recommended cases/All participating patients) or % (Number of accepted cases/Number of recommended cases)

Data were analyzed using the chi-squared test

### Process measurements

**Fig. 1:**
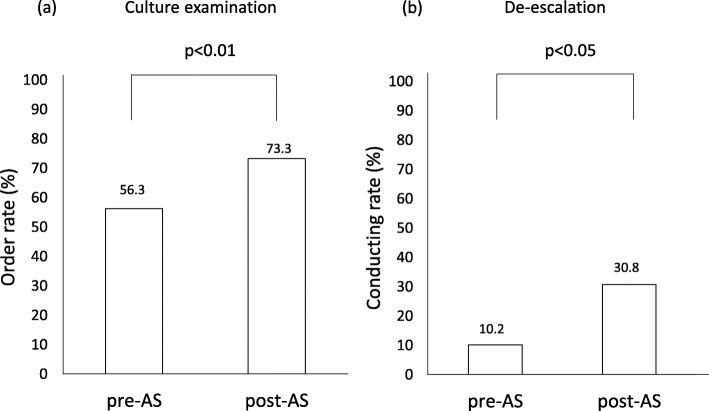
Comparison of the order rate of culture examinations **a** and the conducting rate of de-escalation **b** between pre- and post-AS periods. The chi-squared test was used for statistical analyses

The order rate of culture examinations for the identification of microbial pathogens and the conducting rate of de-escalation are shown in Fig. 1. The order rate of culture examinations significantly increased (*p* < 0.01) from 56.3% in the pre-AS period to 73.3% in the post-AS period. The conducting rate of de-escalation significantly increased (*p* < 0.05) from 10.2% in the pre-AS period to 30.8% in the post-AS period.

AUD, DOT, and the AUD/DOT ratio of carbapenems (MEPM and IPM/CS) and TAZ/PIPC in both periods are shown in Table 4. The AUD of carbapenem significantly decreased (*p* < 0.05) from 8.28 ± 3.39 in the pre-AS period to 3.67 ± 1.73 in the post-AS period. The AUD of TAZ/PIPC also significantly decreased (*p* < 0.05) from 15.05 ± 5.91 to 8.55 ± 3.47. The DOT of carbapenems significantly decreased (*p* < 0.01) from 21.62 ± 7.23 in the pre-AS period to 5.88 ± 1.76 in the post-AS period. The DOT of TAZ/PIPC also significantly decreased (p < 0.05) from 20.93 ± 8.76 to 12.65 ± 4.96. The AUD/DOT ratio of carbapenem significantly increased (p < 0.01) from 0.37 ± 0.07 in the pre-AS period to 0.60 ± 0.12 in the post-AS period, while that of TAZ/PIPC decreased from 0.73 ± 0.08 to 0.67 ± 0.07, which was not significant.

**Table 4:** Changes in AUD, DOT, and the AUD/DOT ratio between pre- and post-AS periods

	Carbapenem			TAZ/PIPC		
	pre-AS	post-AS	*p*-value	pre-AS	post-AS	*p*-value
AUD	8.28 ± 3.39	3.67 ± 1.73	< 0.05	15.05 ± 5.91	8.55 ± 3.47	< 0.05
DOT	21.62 ± 7.23	5.88 ± 1.76	< 0.01	20.93 ± 8.76	12.65 ± 4.96	< 0.05
AUD/DOT	0.37 ± 0.07	0.60 ± 0.12	< 0.01	0.73 ± 0.08	0.67 ± 0.07	0.15

Values are presented as means±SD

*AUD*: Antimicrobial use density (DDDs/1000 patient-days), *DOT*: Days of therapy (DOTs/1000 patient-days)

Data were analyzed using the Mann-Whitney U-test

### Clinical outcomes

**Fig. 2:**
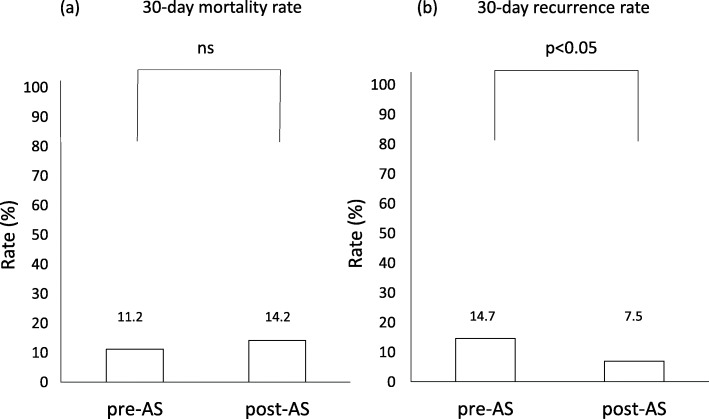
Comparison of the 30-day mortality rate **a** and 30-day recurrence rate **b** between pre- and post-AS periods. The chi-squared test was used for statistical analyses

Thirty-day mortality and recurrence rates in the pre- and post-AS periods are shown in Fig. 2. Thirty-day mortality rates in the pre- and post-AS periods were 11.2 and 14.2%, respectively, and were not statistically different. Thirty-day recurrence rates were 14.7 and 7.5%, respectively, which was significantly (*p* < 0.05) lower in the post-AS period than that in the pre-AS period.

As described above, a decreases were observed in the AUD and DOT of carbapenem and TAZ/PIPC in the present study. Based on these results, improvements in the appropriate use of antimicrobials were achieved without negatively affecting clinical outcomes by AS in this hospital.

## Discussion

Small- to medium-sized hospitals have finite medical resources. Therefore, the implementation of ASPs has to be performed by selecting items available, and their impact may be limited. An assessment of the impact of implementing ASPs is important. Mas-Morey et al. evaluated 28 studies implemented in 26 hospitals with < 500 beds and reported that interventions were not generally associated with significant changes in mortality or readmission rates; however, substantial cost savings were achieved, mainly due to the reduced use of antibiotics or the administration of cheaper antibiotics [10]. The present study compared the use of antimicrobials and its clinical outcomes between the pre- and post-AS periods at a 126-bed community hospital. The results obtained showed that the appropriate use of carbapenem and TAZ/PIPC improved and the 30-day recurrence rate significantly decreased; however, the 30-day mortality rate did not significantly change. Therefore, the implementation of AS in this hospital appeared to effectively improve the appropriate use of antimicrobials, similar to or more effectively than that reported for other hospitals.

The strategy of this study for ASP was to implement a prospective audit and feedback by AST closely communicating with ward pharmacists and pharmacists working on post-prescription audits. Dallit et al. highlighted 2 important core strategies for improving the impact of implementing ASPs: 1) a prospective audit with an intervention and feedback and 2) formulary restriction with preauthorization [6]. Anderson et al. recently reported that a post-prescription audit and review were a more feasible and effective strategy for AS in settings with limited resources and expertise than preauthorization [11]. Furthermore, Muraki indicated that a prospective audit and feedback were realistic for improving the implementation of AS in Japan, and close communication between the AST pharmacist and ward pharmacists was important for consecutive support of this implementation [14]. Doenberg et al. reported that the introduction of a weekly prospective audit and feedback was insufficient for effective ASP implementation, but achieved modest decreases in the utilization of antibiotics [15]. These findings are considered to support the strategy of the present study.

The present results showed that the recommendation rate to prescribers from AST significantly increased from 10.4 to 21.1% (Table 3), and the order rate of culture examinations also significantly increased from 56.3 to 73.3% (Fig. 1a). These increases are considered to be a direct impact of early prospective audits and feedback to prescribers from AST and are attributed to close communication between AST and pharmacists, as described above.

The conducting rate of de-escalation significantly increased from 10.2% in the pre-AS period to 30.8% in the post-AS period (Fig. 1b). Culture examinations are necessary for the appropriate selection of antibiotics, including the implementation of de-escalation [16]. This increase was attributed to the significantly higher order rate of culture examinations. On the other hand, the order rate of culture examinations was 73.3%, indicating that examinations were not ordered for the other 26.7% cases in the post-AS period, which is an issue that needs to be resolved. One approach to achieve improvements is to educate medical staff, including prescribers, about the importance of culture examinations through closer communication.

Decreases were observed in the AUD and DOT of carbapenem and TAZ/PIPC, respectively (Table 4), which indicated a reduction in the amount used and a shorter period of use of these antibiotics. The decreases observed in AUD of carbapenem and TAZ/PIPC in the present study were consistent with previous findings [4]. The decrease in the DOT of carbapenem was also consistent with previous findings [5, 17], whereas that of TAZ/PIPC was not. Yamada et al., [5] reported that the DOT of TAZ/PIPC significantly increased in the post-ASP period, which may be attributed to the use of TAZ/PIPC as an alternative to carbapenems for severe infections and those with long-term antibiotic use. In the present study, the decrease in the DOT of TAZ/PIPC was mainly due to an increase in the conducting rate of de-escalation, and carbapenems and TAZ/PIPC were both typically switched to sulbactam/ampicillin or third-generation cepharosporin. This appears to have been the reason for the differences between the present results and previous findings.

The AUD/DOT ratio of carbapenem significantly increased (Table 4). Carbapenems are some of the most important antibiotics for the treatment of severe or intractable infectious diseases. The daily dose of carbapenem was recently increased according to the worldwide recognition of the pharmacokinetic/pharmacodynamic (PK/PD) theory. Regarding MEPM, the administration of a daily dose of more than 3 g based on the PK/PD theory was reportedly safe and achieved more beneficial effects [18]. However, in this hospital, a daily dose of 1 g was generally administered according to the instructions on the package insert of medicines for common infectious diseases in the pre-AS period. In the present study, the ratio increased to close to 1.0, which may be attributed to closer communication and better feedback between AST and prescribers, including recommendations based on therapeutic guidelines for infectious diseases [19, 20]. On the other hand, the AUD/DOT ratio of TAZ/PIPC did not significantly change. The average value of 0.73 in the pre-AS period was considered to be high because it was close to 1.0 and was maintained in the post-AS period.

In the present study, a significant decrease was observed in the 30-day recurrence rate; however, in a review of 28 studies, Mas-Morey et al. reported no significant reductions in readmission rates after the implementation of ASP [10]. The mediator(s) for the significant decrease in the recurrence rate in the present study remain unclear. One possibility may be longer hospital stays in the present study than in the studies reviewed by Mas-Morey et al. [10]. Average hospital stays in the present study were 38.2 days in the pre-AS period and 32.0 days in the post-AS period. In contrast, average or median hospital stays in the pre- and post-AS periods of some of the studies reviewed by Mas-Morey et al. were 3.40 and 2.69 days [21], 13.2 and 10.8 days [22], and 9.0 and 5.7 days [23], respectively. The longer continuous implementation of post-prescription audits and feedback to prescribers by pharmacists working on post-prescription audits in addition to careful checks of patients’ conditions by ward pharmacists may have contributed to the decrease observed in the 30-day recurrence rate.

The present study had the following limitation: the relationship between clinical outcomes and AS was not clarified. Therefore, further progress in AS activity is necessary to improve clinical outcomes.

## Conclusions

The implementation of AS at this 126-bed community hospital significantly increased recommendations on the appropriate use of antimicrobials from AST to prescribers. It also increased the order rate of culture examinations and the conducting rate of de-escalation. Furthermore, decreases were observed in the amount and periods of use of carbapenems and TAZ/PIPC without a negative impact on clinical outcomes, such as 30-day mortality and recurrence rates, in the post-AS period from the pre-AS period. The beneficial effects of AS are considered to be achieved by close communication between pharmacists working on post-prescription audits and pharmaceutical care at the bedside and AST.

## Data Availability

The datasets generated and/or analyzed during the current study are not publicly available because the transfer of data containing personal information outside of Tarumizu Chuo Hospital is not allowed.

## Abbreviations

AMR: Antimicrobial resistance
AS: Antimicrobial stewardship
AUD: Antimicrobial use density
DOT: Days of therapy
AST: Antimicrobial stewardship team
ASP: Antimicrobial stewardship program
ICT: Infection control team
ICD: Infection control doctor
TAZ/PIPC: Tazobactam-Piperacillin
MEPM: Meropenem
IPM/CS: Imipenem/cilastatin
DDD: Defined daily dose
PK/PD: Pharmacokinetic/pharmacodynamics
